# Anatomical Mechanisms of Leaf Blade Morphogenesis in *Sasaella kogasensis* ‘Aureostriatus’

**DOI:** 10.3390/plants13030332

**Published:** 2024-01-23

**Authors:** Wanqi Zhao, Zhuo Lv, Hanjiao Zhang, Jiahui Yue, Xu Zhang, Long Li, Feiyi Huang, Shuyan Lin

**Affiliations:** 1 Co-Innovation Center for Sustainable Forestry in Southern China, Nanjing Forestry University, Nanjing 210037, China; wanqzhao@163.com (W.Z.);; 2 Bamboo Research Institute, Nanjing Forestry University, Nanjing 210037, China; 3 College of Life Sciences, Nanjing Forestry University, Nanjing 210037, China

**Keywords:** *Sasaella kogasensis* ‘Aureostriatus’, leaf morphological characteristics, anisotropy, rapid elongation, cell division, cell expansion

## Abstract

There are limited studies on the cytology of bamboo leaf development from primordium to maturity. This study delves into the leaf morphological characteristics and growth patterns of *Sasaella kogasensis* ‘Aureostriatus’ and provides a three-dimensional anatomical analysis of cell division, expansion, and degradation. Leaves on the same branch develop bottom-up, while individual leaves develop the other way around. Like bamboo shoots and culms, the leaves follow a “slow–fast–slow” growth pattern, with longitudinal growth being predominant during their development. The growth zones of individual leaves included division, elongation, and maturation zones based on the distribution of growth space. By measuring 13,303 epidermal long cells and 3293 mesophyll cells in longitudinal sections of rapidly elongating leaves, we observed that in the rapid elongation phase (S4–S5), the division zone was located in the 1–2 cm segment at the bottom of the leaf blade and maintained a constant size, continuously providing new cells for leaf elongation, whereas in the late rapid elongation phase (S6), when the length of the leaf blade was approaching that of a mature leaf, its cells at the bottom of the blade no longer divided and were replaced by the ability to elongate. Furthermore, to gain an insight into the dynamic changes in the growth of the *S. kogasensis* ‘Aureostriatus’ leaves in the lateral and periclinal directions, the width and thickness of 1459 epidermal and 2719 mesophyll cells were counted in the mid-cross section of leaves at different developmental stages. The results showed that during the early stages of development (S1–S3), young leaves maintained vigorous division in the lateral direction, while periplasmic division gradually expanded from the bottom to the top of the leaf blade and the number of cell layers stabilized at S4. The meristematic tissues on both sides of the leaf were still able to divide at S4 but the frequency of the division gradually decreased, while cell division and expansion occurred simultaneously between the veins. At S6, the cells at the leaf margins and between the veins were completely differentiated and the width of the leaf blade no longer expanded. These findings revealed changes in cell growth anisotropically during the leaf development of *S. kogasensis* ‘Aureostriatus’ and demonstrated that leaf elongation was closely related to the longitudinal expansion of epidermal cells and proliferative growth of mesophyll cells, whereas the cell division of meristematic tissues and expansion of post-divisional cells contributed to the increases in blade width and thickness. The presented framework will facilitate a further exploration of the molecular regulatory mechanisms of leaf development in *S. kogasensis* ‘Aureostriatus’ and provide relevant information for developmental and taxonomic studies of bamboo plants.

## 1. Introduction

In contrast to animals, plant development is characterized by the continuous and repeated formation of new structures and organs until the life cycle ends. The development of plant organs is an iterative process that includes organ localization, cell type determination, and organ morphogenesis. Morphogenesis encompasses the origin, development, and completion of the external form and internal structure. The leaf is an essential plant organ for photosynthesis, and leaf morphogenesis is critical to plant morphology and yield production [1]. The fundamental components of plant leaves are the leaf base, petiole, and blade, with the latter consisting of the epidermis, mesophyll (mainly composed of the palisade and spongy tissues), and veins. Although the leaf structure is relatively simple, its development involves several specific stages, including leaf initiation, polarity determination, cell division, expansion, and pattern formation, which are precisely coordinated in time and space by multiple pathways, including cell physiology, transcription factors, phytohormones, and miRNAs [2,3,4,5,6,7,8,9,10]. Therefore, comprehending the process of leaf morphogenesis is crucial to grasping plant ontogenesis.

The initiation of aboveground organ development is rooted in the shoot apical meristem (SAM), whose primary purpose is to generate a set of cells capable of differentiating into a stem, leaf, or flower [11,12]. The cells of the ground meristem, located at the center of the SAM, divide at a relatively low rate and remain undifferentiated [13,14,15]. In contrast, the protodermal cells located in the periphery of the SAM divide at a faster rate and in an orderly manner, protruding from the surface of the SAM to form a primordium [14,16]. Throughout the development of the leaf primordium, directional cell divisions establish three significant spatial axes, specifically the adaxial–abaxial axis, the medial–lateral axis, and the proximal–distal axis, after which the primordium grows and differentiates along the growth axes based on this foundation [17]. The transition from leaf primordia to fully developed leaves is a dynamic process propelled by cellular division and elongation [18,19]. The research on leaf growth has focused primarily on the developmental timetable of the abaxial epidermis, with growth zones defined based on cell status. During the initial stages of leaf elongation, the entire leaf is considered a division-active region. The proliferative activity of the cells involves the continuous production of small, uniformly sized dividing cells to maintain leaf growth. Cell division first ceases at the leaf tip, then most cells gradually exit the cell cycle and expand towards the leaf base. After cell division ceases in the developing leaf, cell proliferation is limited to a meristem-like region at the leaf blade base. Further growth in the rest of the leaf is mainly achieved through cell expansion [18,20,21,22,23]. However, studies of *Arabidopsis thaliana* (L.) Heyhn. leaves during the early stages of development have shown that the transition from cell proliferation to expansion occurs abruptly rather than gradually along the leaf blade towards the base [23].

Many previous studies have used kinematic methods to meticulously characterize the spatiotemporal heterogeneity of cell division and expansion during leaf development. Two genes regulating polarity-specific leaf expansion, *AN* (*ANGUSTIFOLIA*) and *ROT3* (*ROTUNDIFOLIA3*), have been identified in *A. thaliana* [24]. The expression of *ROT3* in leaves increased gradually from the proliferative stage to the maturation stage. This coincided with significantly faster longitudinal elongation than lateral growth, suggesting that *ROT3* positively affects longitudinal growth. Conversely, the expression pattern of *AN* was opposite to that of *ROT3* [25,26]. Furthermore, elevated levels of *SPLs* (*squamosa promoter binding protein-likes*) promoted anisotropy in epidermal cell growth on the abaxial of *A. thaliana* leaves, while the expansion of cell growth anisotropy, as well as the prolongation of the proliferation period of cells at the base of the leaf, resulted in leaf expansion and longitudinal expansion, leading to the establishment of mature leaf morphology [27]. In monocotyledons, leaf growth occurs predominantly by longitudinal elongation, which is the main factor contributing to the leaves’ elongated and flattened shape [28,29,30,31,32,33]. Only a limited number of studies have shown anisotropy in leaf growth. Studies of leaf shape in tall fescues have shown that as the intercalary meristem develops, expansion in width and thickness occurs mainly at the leaf blade base, whereas relative longitudinal elongation away from the leaf base predominates [21]. A model of leaf architecture was constructed in *Oryza sativa* L. based on morphological changes in different plant leaves. The study showed that the size of the potential leaf is predetermined at the onset of leaf growth [32]. In a kinematic analysis of the expansion of maize leaves along the three growth axes, longitudinal growth was consistently dominant, while treatment with gibberellins increased the growth anisotropy by specifically stimulating the rate of longitudinal growth but did not affect transversal and adaxial–abaxial expansion [33].

Bamboo is characterized by rapid growth, adaptability, and versatility, and the plants are of immense value in basic research and production practice. Bamboo’s structure includes shoots, culms, root systems, flowers, and fruits [34,35,36,37,38,39,40,41,42]. The leaves constitute crucial source organs of bamboo, and mature leaves comprise leaf sheaths, ligules, auricles, pseudopetioles, and blades [43]. Plants can perform diverse functions through the precise spatial categorization of specific cell types. The anatomical structure of bamboo leaves includes the adaxial and abaxial epidermises, mesophyll, and a network of veins. Most of the existing literature on bamboo leaves focuses on their anatomy, chemical composition, and biological properties [44,45,46,47]. However, to date, only a few studies have investigated the development of bamboo leaves. Su et al. [48] constructed three-dimensional structural maps of the anatomies of leaves from six bamboo species. The abaxial epidermis of linearly growing leaves of *Pseudosasa japonica* was observed to have a division zone of approximately 10 mm, an elongation zone of approximately 29 mm, and a maturation zone, and a precise developmental cue for the abaxial epidermis was identified [49,50]. The publication of the *Phyllostachys edulis* genome sequence in 2018 has led to the unveiling of many genes involved in bamboo development, and the development of high-throughput technologies is further improving our understanding of the regulation of leaf growth in bamboo plants [51,52]. In *P. japonica* leaves, transcripts for cell division have been changed to those for cell elongation, photosynthetic development, secondary metabolism, stress tolerance, and nutrient transport to the distal part of the leaf [49]. A variant of *P. japonica*, known as *P. japonica* var. *tsutsumiana*, was identified as having abnormal stomatal development, and subsequent validation showed *dlt* (*dwarf and low-tillering*) as a key factor in regulating the stomatal density in rice and bamboo. However, the cytology of the development of bamboo leaves from small leaf primordia into mature leaves remains unreported.

In this investigation into the anatomical mechanism of leaf development in *S. kogasensis* ‘Aureostriatus’, we analyzed the three-dimensional growth process of leaf blades at various developmental stages using the paraffin sectioning technique. Here, we analyzed the morphological transition from the leaf primordium to mature leaf by measuring the size and shape of epidermal and mesophyll cells and identified critical transition points during leaf development at the cellular level. Our results advance our comprehension of the cytological mechanisms of morphogenesis in bamboo plants and provide insight into taxonomic and developmental investigations of bamboo plants.

## 2. Results

### 2.1. Morphological Characteristics of Leaf Blade Development of S. kogasensis ‘Aureostriatus’

**Figure 1:**
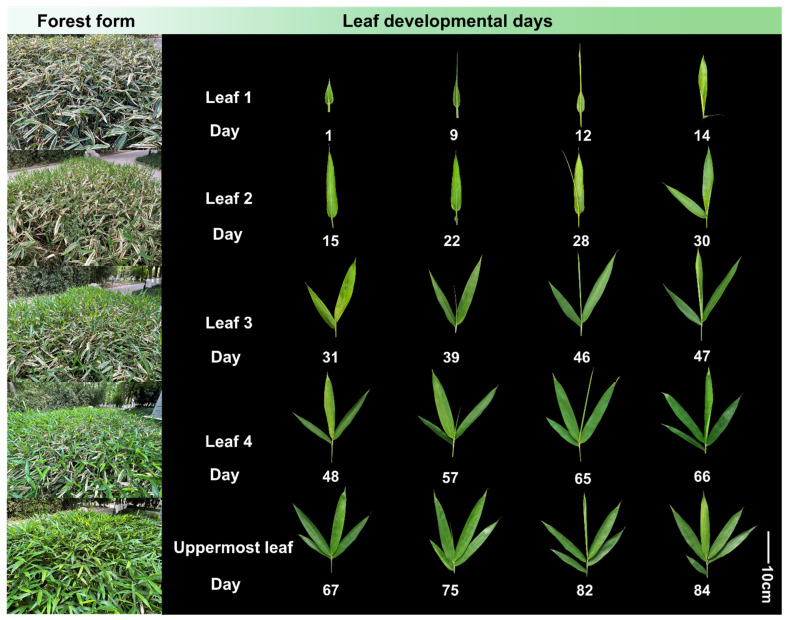
Stand characteristics and leaf blade development during the period from 1 to 84 days in *S. kogasensis* ‘Aureostriatus’.

On average, a leaf takes 15 days to reach full maturity. Leaves show a systematic upward growth trend, following a structured developmental pattern; as the apex of one leaf unfolds, the next leaf emerges from the sheath. Extending leaves from tightly wrapped sheaths typically take longer but unfurling of fully rolled leaves only require 1–2 days (Figure 1).

**Figure 2:**
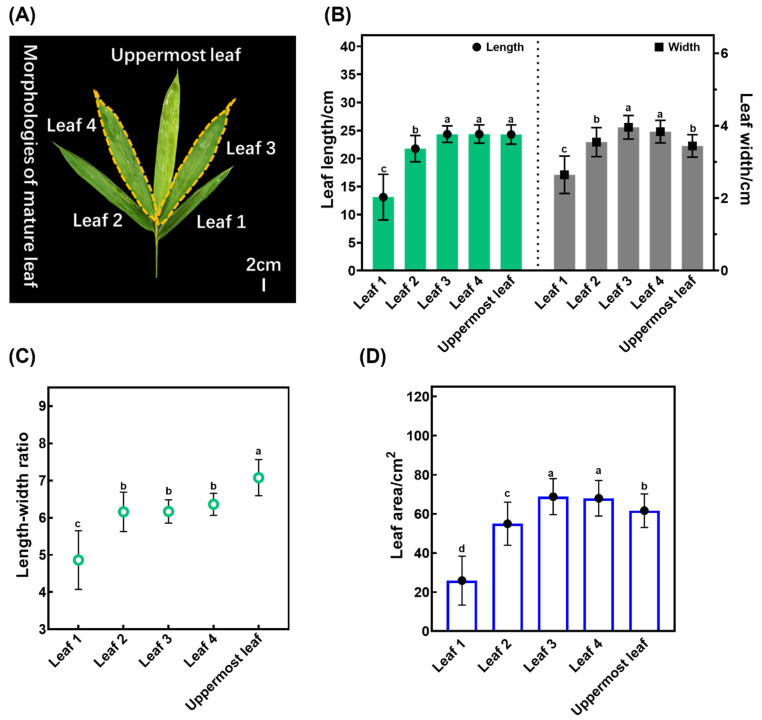
An analysis of the size of leaf blades in *S. kogasensis* ‘Aureostriatus’: (**A**) the morphology of all leaves on a single branch; (**B**) the length and width of every leaf blade on the branch; (**C**) the ratio of the leaf blade length to width; (**D**) the area of the leaf blade. Different letters in the graph indicate significant differences at a level of *p* < 0.05.

To investigate the growth and development of individual leaf blades, we first conducted separate measurements of the morphological indices of each leaf on the branch. These measurements included the length (Figure 2B), width (Figure 2B), leaf area (Figure 2C), and length–width ratio (Figure 2D). However, the leaves occupying the third and fourth positions on the branch showed greater uniformity. The leaf at the top of the branch had significantly higher aspect ratio values than its counterparts, indicating a more elongated shape than the other leaves. Our results suggest that the third and fourth leaves showed the most stable growth, making them ideal as representatives for subsequent research on growth and development (Figure 2A).

### 2.2. Three-Dimensional Morphological Characterization of S. kogasensis ‘Aureostriatus’ Leaf Blades

**Figure 3:**
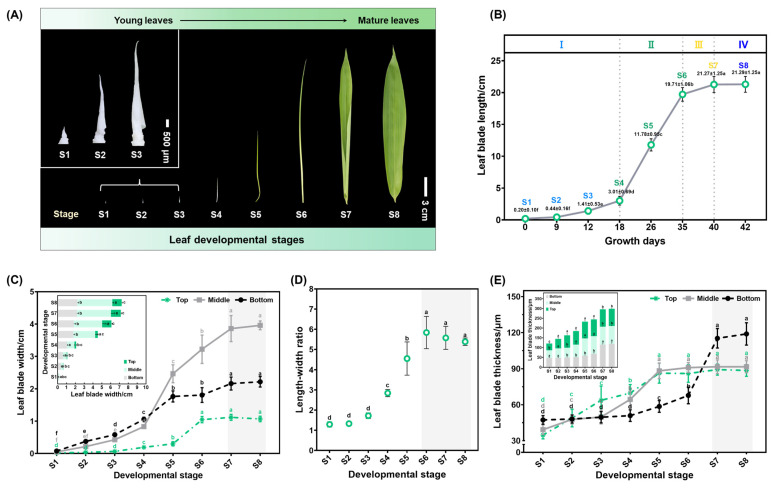
Leaf blade growth in three dimensions of *S. kogasensis* ‘Aureostriatus’. (**A**) The process of growth from leaf primordia to functional leaves. (**B**) The growth trend of leaf elongation displays a “slow–fast–slow” pattern and comprises of four distinct phases: (I) the early elongation phase (S1–S3); (II) the rapid elongation phase (S4–S6); (III) the slow elongation phase (S6,S7); (IV) the maturation phase (S7,S8). (**C**) Variations in leaf width at different points during elongation, changes in length-to-width ratios in leaf blades (**D**), and fluctuations in leaf thickness during elongation (**E**). Different letters in the graph indicate significant differences at a level of *p* < 0.05.

The structure of mature monocot leaves is determined by three growth axes—adaxial–abaxial (thickness), medial–lateral (width), and proximal–distal (length). The flattened and narrow morphology of bamboo leaves indicates notable differences among their three-dimensional axes. The leaf dimensions were measured during the 42 days of the single leaf development of *S. kogasensis* ‘Aureostriatus’ to examine how cell growth is coordinated across three growth axes. The data provided information on the transition from the young primordium morphology to mature leaves. It was evident that the leaf growth and development follow a basipetal sequence, as depicted in Figure 3A. Our investigation identified eight pivotal time points in leaf growth, which are categorized into four stages—initiation (S1), pre-appearance (S2–S4), post-appearance (S5–S6), and maturity (S7–S8) (Figure 3A). The length of the leaf increased from 0.20 ± 0.10 cm (standard error of the mean; SEM) to 21.29 ± 1.25 cm (SEM) during this period. The developmental stages were reclassified by measuring the leaf length, as demonstrated in Figure 3B. During the early elongation stage (S1–S3), when the lengths of the leaf blades ranged from 0.20 ± 0.10 cm to 1.41 ± 0.53 cm (SEM), the young leaves grew slowly for 12 days. However, from day 18, we observed that the leaves elongated rapidly. During the rapid elongation stage (S4–S6), the length of the leaves increased from 3.01 ± 0.69 cm (SEM) to 19.71 ± 1.06 cm (SEM) at a growth rate of 0.93 cm/d. When the leaf blade was approaching its final length, it entered the stage of slow elongation (S6–S7), which marked the beginning of leaf growth cessation, and the growth length of the leaf blade was less than 2 cm in 5 days. The development of the leaf blade proceeded until the 40th day, when it entered the maturity stage (S7–S8) and reached a length of 21.27 ± 1.25 cm (SEM), like the functional leaf, after which growth and development ceased until the leaf blade was completely unfolded two days later.

In the early elongation stage (S1–S3), the blade length-to-width ratios of young leaves were 1.29, 1.33, and 1.72, respectively, suggesting approximately equal lengths and widths of the blade at this stage (Figure 3D). As the blade lengths grew slowly, the leaf blade widths also increased sluggishly during the initial stage (S1–S3). However, after S4, the longitudinal elongation exceeded the transverse elongation, resulting in an upward trend in the leaf blade length/width ratio from 2.84 to 5.83. After stage 6, the growth of the leaf width predominated, as indicated by a reduction in the ratio, and ultimately the leaf size became constant after stages 7 and 8. The widths of various portions of the leaf blade exhibited variability during development. A gradual decline in width from the base to the tip of the blade characterizes S1–S4. However, after S4, the width of the central portion of the blade increases significantly, culminating in maximum width during S7 and S8. At S6, there is a cessation of expansion of the apical width, but the central and base parts of the leaf are still expanding. The maximum width of the leaf was noted on day 40, coinciding with the completion of leaf blade growth and reaching the final size (Figure 3C).

The leaf thicknesses varied at different sites during development (Figure 3E). Like the width, the leaf thickness tended to increase during leaf elongation at all sites. The blade thickness was non-uniform in different portions of the individual leaves. The basal part was thickest at S1, gradually decreasing acropetally, and all parts had the same thickness at S2, with the upper and middle parts thickening gradually after S2 and stabilizing after S5. The base’s thickness remained stable until S4 and then gradually increased from S5 to S6. The leaf length and width growth stabilized after stage 6, while the thickness at the base of the leaf significantly increased and remained stable at S7–S8, with the maximum thickness shifting towards the leaf base.

### 2.3. Anatomical Characteristics of Leaves of S. kogasensis ‘Aureostriatus’ during Rapid Elongation

#### 2.3.1. Division and Differentiation of Epidermal Cells during Rapid Elongation

The epidermis comprises short and long cells, stomatal complexes, and prickly hairs, with the long cells being the most numerous cell types. To enhance the observation of the elongation of epidermal cells, we chose leaf samples during rapid elongation (S4–S6) and studied the development of long cells in both adaxial and abaxial epidermises in longitudinal sections. The linearly arranged long cells were classified into three categories—long cells on the adaxial and abaxial epidermises (Ad/Ab-LC) and in the stomatal rows (LCS).

**Figure 4:**
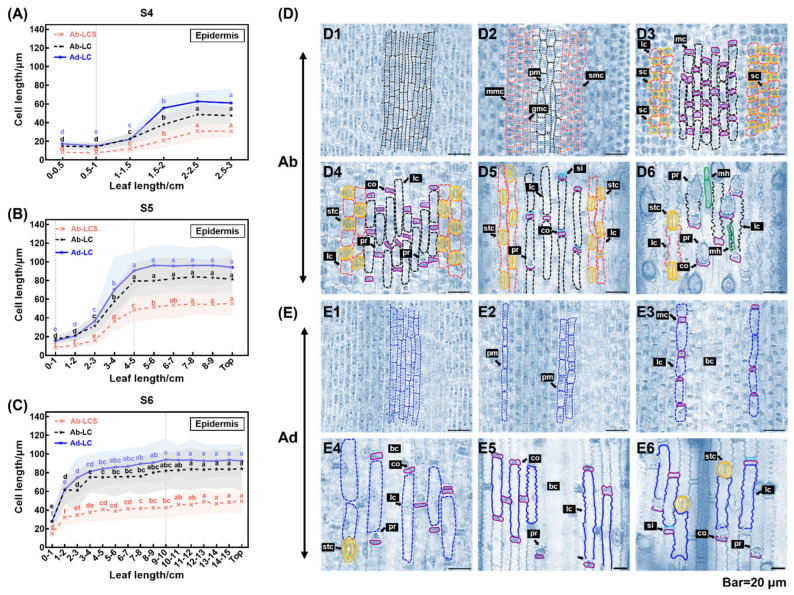
Roadmap of epidermal cell development during rapid elongation stage. (**A**–**C**) Lengths of three types of long cells in the proximal–distal axial plane of leaves of different lengths during rapid elongation. The developmental roadmaps of the (**D1**–**D6**) abaxial and (**E1**–**E6**) adaxial epidermises during rapid elongation are illustrated. Scale bars are 20 μm. Note: ab, abaxial epidermis; Ab-LC, long cells on the abaxial epidermis; ad, adaxial epidermis; Ad-LC, long cells in the stomatal rows; Ad-LCS, long cells on the adaxial epidermis; bc, bulliform cells; co, cork cell; gmc, guard mother cell; lc, long cell; mc, meristemoid cell; mh, micro-hair; mmc, meristemoid mother cell; pm, protodermal cell; pr, prickle hair; sc, subsidiary cell; si, silica cell; smc, subsidiary mother cell; stc, stomatal complexes; (**A**–**C**) indicate significant differences at a level of *p* < 0.05 with different letters.

Figure 4 illustrates the use of the length of epidermal cells in anatomical structures to delineate the growth zones of rapidly elongating leaves. A total of 13,303 long cells were measured at different developmental stages, revealing a shift in cell dimensions across various growth zones during leaf elongation. During the early phase of quick elongation (S4), cells within the 1 cm region at the leaf base demonstrated a small size and a steady average length. These cells exhibited considerable mitotic activity, indicating that their principal role was to undergo proliferation (Figure 4A,D1,D2). Conversely, cells distal to the 1 cm basal region exhibited less rounded shapes, increased sizes, and gradual elongation, suggesting that cell elongation was the primary growth mode. Similarly, in S5, the 1 cm region at the base of the leaf consisted entirely of proliferating cells but no mitoses were found in the more distal leaf portions; however, the increased cell expansion capacity extended to the middle of the leaf (Figure 4B). In S6, we observed that the whole leaf was no longer proliferative and the cells of the leaf blade elongated from the leaf base along the developmental axis, with a reduced inclination to elongate towards the middle of the leaf (Figure 4C). However, in S5 and S6, from the leaf’s center to its top, Ab-LC and Ad-LC complete their elongation and have comparable cell lengths, while LCS are still elongating and are evidently shorter in comparison to the other two long cell types (Figure 4B,C). The zone of division for leaves experiencing rapid elongation was identified within a 1 cm section of the leaf’s foundation in S4 and S5, representing 33% and 8% of the proximal–distal axis, respectively. The elongation zone was distributed across S4–S6, with roughly 2, 4, and 10 cm lengths in S4, S5, and S6, respectively (Figure 4A,C). The section of the blade spanning from the middle to the tip of the leaf in S5 and S6 was the zone of maturation (Figure 4D5,D6).

The study determined the developmental processes of epidermal cells. Protodermal cells were present in the division zone within 0.5 cm from the leaf base (Figure 4D1,E1). Among the three types of long cells, only the development of LCS (long cells in the stomatal rows) was linked to stomatal developmental processes. Rows of small, square meristematic histoblasts were observed at distances of 0.5–1 cm from the leaf base. These structures were formed by divisions of a group of protodermal cells located on the abaxial epidermis (Figure 4D2). The small square cell underwent a single asymmetric division, resulting in a daughter and guard mother cells, which later developed into the LCS, as depicted in Figure 4D2. At a distance of 1–2 cm from the base of the leaf blade, the subsidiary mother cell underwent division, resulting in two cells of unequal size—a small subsidiary cell and a long cell, as shown in Figure 4D3. The guard mother cell and two subsidiary cells (which originated from the division of the subsidiary mother cell on either side) formed a stomatal complex in a 2–3 cm region at the base of the leaf blade (Figure 4D4). At a distance of 3–4 cm from the base of the leaf blade, the subsidiary cell expanded and surrounded the guard mother cell, creating a gap in the middle of the structure (Figure 4D5). During stomatal differentiation, a significant elongation of the LCS was observed (Figure 4D3–D5). In the mature leaf epidermis, the edge of the LCS forms a serrated protrusion that allows it to be tightly connected to the other LCSs and the stomatal complex (Figure 4D6). The long cells of the non-stomatal rows developed similarly to the adaxial long cells and originated from the protodermal cell (Figure 4D1,E1). During development, the protodermal cell underwent division to produce a long cell and a short meristemoid cell (Figure 4D2,E2). Subsequently, the elongated cell widened as the meristemoid cell divided to produce a silica cell and the cork cell (Figure 4D3–D5,E3–E5). Bulliform cells were observed in the adaxial epidermis but their developmental process was not visible in longitudinal sections (Figure 4E3–E5). The prickly hairs originated as protrusions on the surfaces of cork cells (Figure 4D4). In the mature epidermis, long cells with serrated edges, spike-like prickle hairs, and micro-hairs were observed; the latter two appeared only in the abaxial epidermis (Figure 4D6,E6).

#### 2.3.2. Division, Expansion, and Degradation of Mesophyll Cells during Rapid Elongation

**Figure 5:**
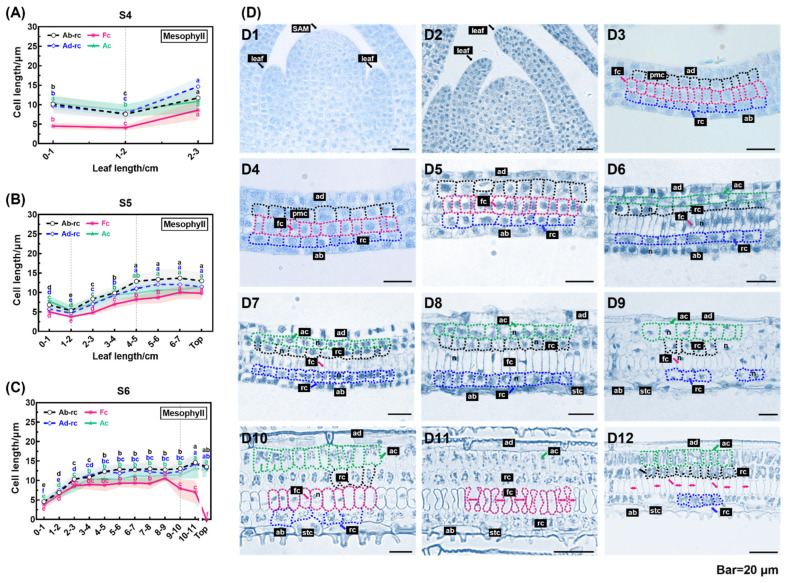
Roadmap of mesophyll cell development during rapid elongation stage: (**A**–**C**) the lengths of four different types of mesophyll cells in leaves of different lengths during rapid elongation. (**D1**–**D12**) structures of the mesophyll tissue of *S. kogasensis* ‘Aureostriatus’ at different stages of development. Scale bars are 20 μm. Note: ab, abaxial epidermis; Ab-LC, long cells on the abaxial epidermis; ac, arm cells; ad, adaxial epidermis; Ad-LC, long cells in the stomatal rows; Ad-LCS, long cells on the adaxial epidermis; fc, fusoid cells; n, nuleus; pmc, primary mesophyll cells; rc, rosette cells; SAM, shoot apical meristem; stc, stomatal complexes; (**A**–**C**) indicate significant differences at a level of *p* < 0.05 with different letters.

The mesophyll cell morphology was analyzed in S4–S6. The measurement of the cell lengths of 3293 mesophylls in longitudinal sections showed a decreasing trend within the 0–2 cm region at the leaf blade base in both S4 and S5, indicating that the cells were dividing (Figure 5A,B). In contrast, the lengths of the mesophyll cells located distally to the basal 2 cm showed an increasing trend, indicating that the cells were no longer dividing and had entered the expansion stage. In S4, S5, and S6, the zones of expanding mesophyll cells were approximately 1 cm, 3 cm, and 10 cm in length, respectively. During the S5 stage, there was no change in the length of mesophyll cells located 5 cm from the base. Additionally, in S6, all cells displayed an increasing trend in length, except for the fusoid cells, which decreased in length towards the tip of the leaf.

To gain more insight into the anatomical development of the mesophyll, the longitudinal growth of mesophyll cells (S1–S8) was determined. Longitudinal sections of stem apical meristems from *S. kogasensis* ‘Aureostriatus’ showed that the leaf primordium consists of actively dividing meristematic cells, which initially protrude laterally from the SAM and then continue to grow apically (Figure 5D1,D2). When the length of the leaf blade was less than 0.5 cm (S1–S2), an examination of the longitudinal sections revealed the adaxial and abaxial protoderms and three layers of ground meristematic cells, all of which had the characteristic features of meristematic cells, with a central nucleus, dense cytoplasm, and strong ability to divide (Figure 5D3,D4). At about 1 cm in length (S3), the ground meristem in the middle of the leaf blade already exhibited a clear difference in the cell division plane between the adaxial and abaxial axes. In Figure 5D5, cells in the ground meristem at the adaxial side began to undergo periclinal divisions, giving rise to two layers of primary mesophyll cells. After S4, the final number of mesophyll cell layers was attained (Figure 5D6). During the rapid elongation of the leaves, mitotic cells were identified in the division zone of both the epidermis and mesophyll (Figure 5D7), whereas no mitotic cells were detected in the elongation zone. Figure 5D7,D8 show that the mesophyll cells significantly outnumbered the epidermal cells, and the latter underwent considerable elongation. The primary mesophyll cells gradually differentiated basipetally as the leaf grew. Based on the cell morphology, four groups of cells were distinct in the mesophyll: two layers of irregular parenchymal cells close to the adaxial epidermis, the arm cells (ac) and the rosette cells (ad-rc), which are derived from the same initials; the rosette cells close to the abaxial epidermis (ab-rc); the long elliptical fusoid cells (fc) located between the two layers of rosette cells (Figure 5D9,D10). At the time of maturity, the mesophyll cells have undergone full differentiation, accompanied by an increased cell content, and the fusoid cells in the middle layer of the longitudinal section of the leaf blade have begun to collapse (Figure 5D11). According to our observations, the degradation of the fusoid cells commences when the leaf blade emerges from the sheath. Subsequently, the fusoid cells assume a gradually decreasing “I” shape in the longitudinal section, with the intercellular space continuously expanding (Figure 5D11,D12).

### 2.4. Anatomical Characteristics of the Medial–Lateral and Adaxial–Abaxial Axes during Development of S. kogasensis ‘Aureostriatus’

**Figure 6:**
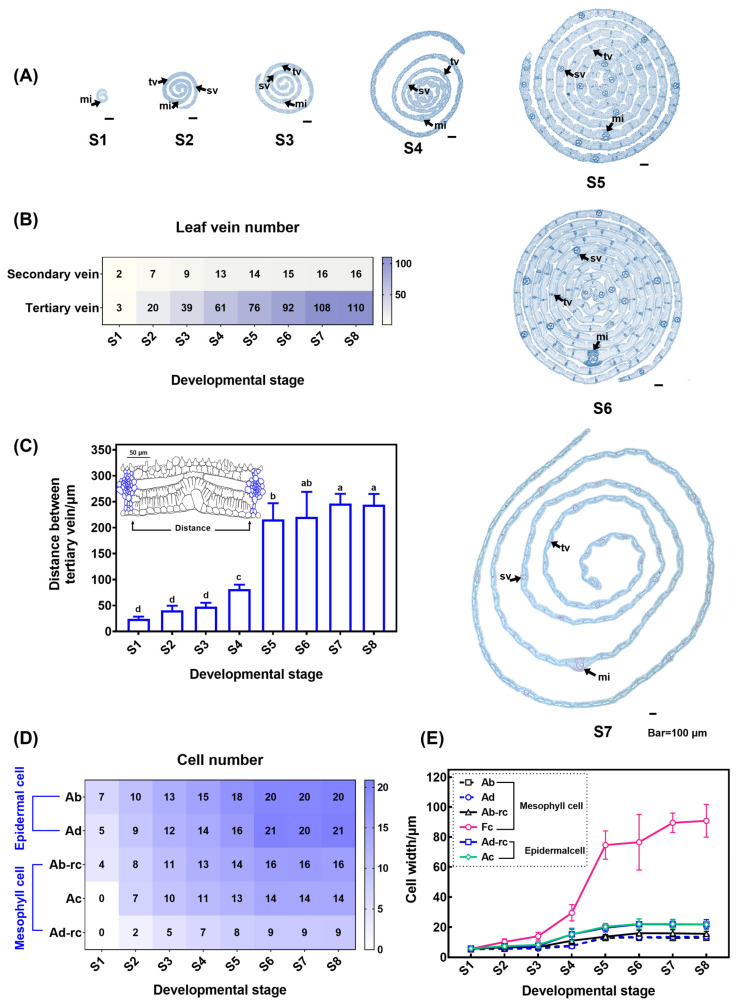
An analysis of leaf width growth of *S. kogasensis* ‘Aureostriatus’: (**A**) transversal sections from the middle of the leaf blade at several developmental stages; (**B**) the numbers of secondary and tertiary veins distributed along the leaf width at various stages; (**C**) the distance between tertiary veins and the number (**D**) and length (**E**) of epidermal and mesophyll cells in the middle of the leaf at various developmental stages. Note: Ab, abaxial epidermis; Ac, arm cells; Ad, adaxial epidermis; Fc, fusoid cells; mi, midrib; rc, rosette cells; sv, secondary vein; tv, tertiary vein; (**C**) shows significant differences at a level of *p* < 0.05, indicated by different letters.

To further analyze the growth pattern of *S. kogasensis* ‘Aureostriatus’ leaves in the medial–lateral direction, we observed the relationship between leaf veins and leaf widths in transverse sections of the middle part of the leaf at different developmental stages. The development of the middle width is displayed in Figure 6A, revealing a close correlation between the total number of leaf veins and the leaf width. The number of veins distributed across the leaf width at different developmental stages was counted. Figure 6B shows that the numbers of secondary and tertiary veins increased as the leaf developed, peaking at the end of leaf growth, with tertiary veins significantly exceeding the secondary veins in number. The average distance between the tertiary veins was measured in the middle of the leaf. No significant difference in vein distance in the early elongation stages (S1–S3) was detected, whereas the distance increased significantly after S4 and reached a maximum at S7 and S8 (Figure 6C). Furthermore, our analysis involved scrutinizing 1459 cells in the epidermis and 2719 cells in the mesophyll adjoining the tertiary veins. Throughout all developmental stages, the number of epidermal cells exceeded that of mesophyll cells. The number of both cell types became constant at S6 and was like that of mature leaves (Figure 6D). The number of distinct fusoid cells in transversal sections remained consistent, with a fixed count of two between the veins. However, their width was noticeably greater than that of the neighboring cells, as shown in the Figure 6E.

**Figure 7:**
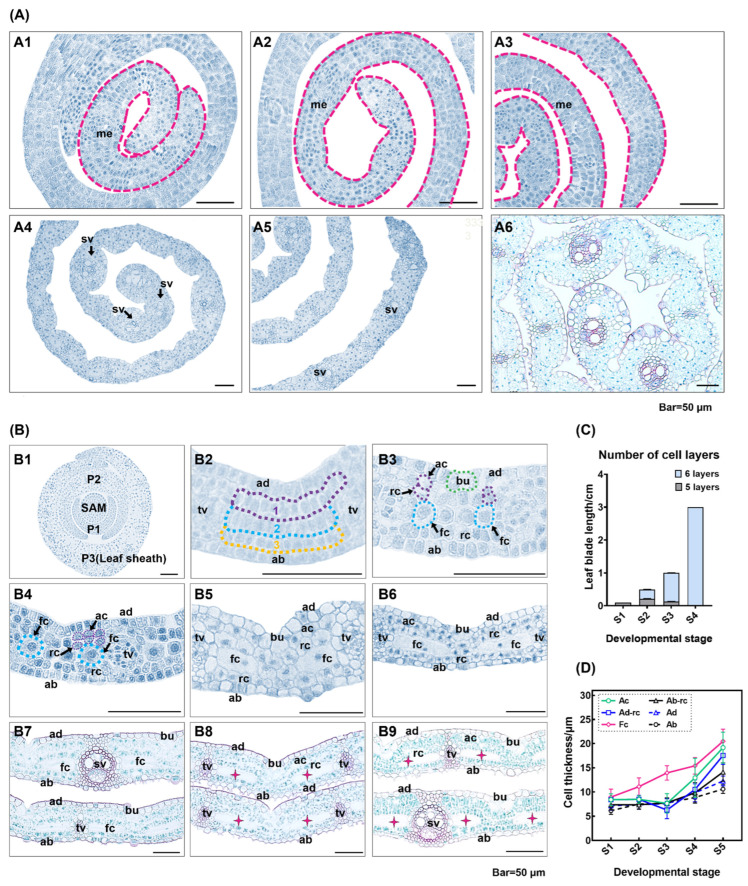
Distribution of the marginal meristem of the leaf middle portion and roadmap of the leaf thickness in *S. kogasensis* ‘Aureostriatus’: (**A1**–**A3**) early in leaf development, young leaves exhibit very distinctive meristem features; (**A4**,**A5**) some cells located between the veins begin to differentiate; (**A6**) the cells located between the margins and veins of the leaf have not yet fully differentiated; (**B1**) structure of leaf primordia in transversal section; (**B2**) at an early stage, a transversal section of a developing leaf blade shows two layers of epidermal cells and three layers of mesophyll cells; (**B3**) cell layers increase as the mesophyll cells adjacent to the adaxial epidermis undergo cell division; (**B4**) the periclinal divisions are complete, increasing the intercellular space and the onset of primary fusoid cell enlargement; (**B5**,**B6**) cell divisions are completed and mesophyll cells continue to enlarge and differentiate; (**B7**,**B8**) gradual degradation of fusoid cells; (**B9**) transverse structure of a functional leaf; (**C**) S1–S4, differentiation of cell layers; (**D**) variations in the thickness of the epidermal and mesophyll cells in the middle of the leaf at different stages of development. The pink stars represent fusoid cells that have degenerated. Scale bars are 50 μm. Note: ab, abaxial epidermis; ac, arm cell; ad, adaxial epidermis; bu, bulliform cell; fc, fusoid cell; me, meristem; rc, rosette cell; sv, secondary vein; tv, tertiary vein.

The marginal meristem activity controls the width of leaf blades. During the initial stages of leaf growth, young leaves exhibited significant meristematic characteristics throughout the entire leaf (Figure 7A1–A3). At stage 4, the meristematic tissues on each side evidence a strong ability to divide, while previously formed cells situated between the tertiary veins maintain some ability to divide. Further growth is mainly gained through cell expansion (Figure 7A4,A5). After stage 7, the cells situated at the margins of the leaf, as well as those positioned between the leaf’s veins, undergo differentiation and exhibit minimal further increase in their width (Figure 7A6).

The study depicts the anatomical development of the leaf in the transverse section (Figure 7B1–B9). Young leaves have five cell layers, including three primary mesophyll cell layers (Figure 7B1–B3). These layers consist of cells of the same diameter that have not yet differentiated (Figure 7B2). Observations of the transversal sections were correlated with obtained from the longitudinal section, where primary mesophyll cells adjacent to the adaxial epidermis underwent periclinal divisions to form two smaller cells, the arm and rosette cells (Figure 7B3). We noted a pattern of division beginning in the cells next to the leaf veins and moving toward the interveinal region. In transverse sections, arm cells are formed from primary mesophyll cells adjacent to bulliform cells through cell expansion, rather than periclinal division (Figure 7B3,B4). After the cessation of cell division, the mesophyll cells enlarged laterally and developed into fusoid cells, which then expanded and disintegrated, resulting in the formation of intercellular spaces in the transverse section, as illustrated in Figure 7B5–B9. At the level of the whole leaf, we observed a gradual decrease in the proportion of cells undergoing periclinal division towards the base. By S4, the number of cell layers was stabilized across the leaf, which is evident in Figure 7C. During leaf development, the mesophyll contributed substantially to the leaf’s total thickness as compared to the epidermal cells. Furthermore, these divisions resulted in a transient downward trend of the arm and rosette cells’ thickness on the adaxial surface from S2 to S3 (Figure 7D).

## 3. Discussion

The process of plant organization is complex, involving cell division, growth, and precise coordination in time and space, culminating in the formation of three-dimensional organs [53]. However, the research on the development of bamboo leaves has fallen behind that of other bamboo plant organs. The dwarf bamboo species *S. kogasensis* ‘Aureostriatus’ has significant ornamental value, and its wide leaves are advantageous for both observation and research. Unfortunately, there is currently no documentation of the growth process of these leaves. Throughout our research, we thoroughly observed and analyzed the structural characteristics of *S. kogasensis* ‘Aureostriatus’ leaf blades, using both morphological and anatomical tools to reveal cytological details along the adaxial–abaxial, medial–lateral, and proximal–distal axes. We established a growth model that provided key cellular information for further investigation into the molecular mechanisms behind the growth of *S. kogasensis* ‘Aureostriatus’ leaf blades.

Throughout the study, we monitored the morphological changes in leaf development over 84 days, using the distances between leaf tips and sheaths as notation points, and created a dynamic landscape depicting the morphological growth of all leaves on the branches of *S. kogasensis* ‘Aureostriatus’ (Figure 1). In agreement with previous research on maize leaves, a uniform growth pattern was observed for all leaves along the branch, measured from leaf tip emergence to when the mature leaf is fully expanded [31]. By employing a morphological analysis to identify the representative leaves, we studied the growth and development of *S. kogasensis* ‘Aureostriatus’. As shown in Figure 3B, the elongation of a single leaf follows a “slow–fast–slow” growth pattern like the growth of bamboo shoots and culms [40,54]. The process of transition from the leaf primordium to the mature form undergoes significant morphological changes, and the features derived from the anatomical structures of mature leaves are static and do not give a complete picture of the important dynamic changes that occur from the microscopic to the macroscopic scale during leaf development [44,48,55,56,57]. Our investigation of the morphology and histology has uncovered this developmental dynamic for the first time. The morphological data indicate that longitudinal growth is the dominant factor in *S. kogasensis* ‘Aureostriatus’ leaf development, consistent with similar findings in maize, ryegrass, tall fescue, and other grass species [28,29,33,58,59].

In poaceans, a persistent spatial gradient comprises the division zone, expansion zone, and mature part of the leaf blade [60]. The growth of leaves depends on the temporal and spatial coordination of cell division and expansion, and the epidermis layer on the abaxial surface is thought to reflect cellular mitotic events during leaf elongation in Poaceae [23,61,62]. During rapid leaf elongation in *S. kogasensis* ‘Aureostriatus’, three growth zones with different cellular behaviors were observed, with the spatial distribution of the cell divisions showing a constant pattern within a given elongation period (S4, S5) (Figure 4 and Figure 5). In the epidermal development, the meristematic zone was limited to approximately 10 mm above the base, which is in line with previously conducted research on rapidly growing *P. japonica* and *B. multiplex* [38,49]. The division zone of the mesophyll cells extended to 0–20 mm from the leaf base and was about twice that of the division zone of the epidermal cells, and a similar phenomenon was reported in tall fescue [63,64]. At the last stage of rapid elongation (S6), the basal meristem loses its ability to divide and mainly increases its leaf length through the expansion of the elongation zone. The present study indicates that the elongation of *S. kogasensis* ‘Aureostriatus’ leaf blades is promoted by the combined effect of the longitudinal expansion of epidermal cells and proliferative growth of mesophyll cells. Additionally, the width and thickness dimensions displayed fluctuations over time, signifying an anisotropic process in leaf growth. The growth patterns seemed to vary among poacean plant species. Tall fescue exhibited continuous growth in both width and thickness during leaf elongation, while maize exhibited greater anisotropy in the cell division zone compared to the elongation zone [21,33,63].

The morphological and anatomical features of trophic organs are crucial in systematic classification studies of bamboo. Among trophic organs, bamboo leaf blades have unique and distinctive anatomical structures. These include the epidermal system, leaf vein structure (midrib and secondary veins), mesophyll cell morphology, number of bulliform cells, fusoid cells, and leaf sheath structure. These characteristics are significant for the taxonomy of bamboo plants [44,65]. Bamboo leaves are distinguished from those of other plants in the Poaceae family by the presence of various mesophyll cells, namely arm, rosette, and fusoid cells, with the latter being considered the most typical cells of the leaves of Bambusoideae [56,66]. In the transverse sections of mature leaves, the fusoid cells were significantly larger than other mesophyll cells and appeared devoid of intracellular contents. In the longitudinal sections, the fusoid cells were ‘I’-shaped with prominent intercellular cavities. The fusoid cells may play a role in supporting the leaf structure [67]. The earliest debate about fusoid cells centered on whether they were ‘cells’ or an intercellular space left by the filling of mesophyll cells during development [65,68]. In Poaceae, fusoid cells are derived from the ground meristem. In *Joinvillea ascendens*, the initial fusoid cells divide to form three or four derivative cells. However, only single fusoid cells enlarged without division in *Rugoloa pilosa* (Sw.) *Zuloaga*, *Aulonemia aristulata*, *Otatea rzedowskiorum*, and *Pharus latifolius* [69]. Two fusoid cells were observed in the mesophyll region between the adjacent vascular bundles, separated by 1–2 rosette cells in transversal sections of leaf blades of *S. kogasensis* ‘Aureostriatus’ (Figure 7B). The size of the fusoid cells expanded significantly more than other mesophyll cells during leaf development (Figure 7D), which was consistent with previous studies on *R. pilosa* [69]. Another striking feature of fusoid cells is that their collapse and death are often observed in mature leaf blades [48]. The lysis of organelles, a phenomenon that may be related to programmed cell death, has been observed in *Gramineae* leaves [69]. During S6, the leaf blade extends out of the sheath, and this is correlated with the gradual collapse of the fusoid cells and enlargement of the intercellular spaces (Figure 5C,D10–D12). In the transverse section, protoplasts undergoing degradation were observed (Figure 7B7,B8).

## 4. Materials and Methods

### 4.1. Plant Materials

*Sasaella kogasensis* ‘Aureostriatus’ was grown at Nanjing Forestry University (32°01′ N, 118°48′ E, 8.9 m altitude). The culms ranged in height from 0.5 to 1 m and 3–4 mm in diameter. The leaves measured between 15 and 22 cm in length and from 1.8 to 3 cm in width. The leaf blades attain a green color at maturity, following which irregular yellow stripes appear.

### 4.2. A Survey of Morphological Indicators of Leaf Developmental Processes in S. kogasensis ‘Aureostriatus’

The leaf development dynamics of *S. kogasensis* ‘Aureostriatus’ was observed for two years on the campus of Nanjing Forestry University. The appearance of subsequent leaves was recorded, starting from the day on which the first leaf emerged from the sheath until the leaf at the top of the branch reached full maturity (leaf spreading and no further change in leaf shape) after 84 days. During the growing season in March, 50 *S. kogasensis* ‘Aureostriatus’ plants were assigned labels and numbers due to their uniform growth and optimum development. Daily leaf length measurements were taken and documented. Leaf blades from the same branch (every branch had five leaves) were collected and transported to the laboratory for scanning using an Hewlett-Packard Scanjet 4850 scanner (Moreno Valley, CA, USA). Image J (x64) software was employed to calculate the leaves’ length, width, and area from the scanned images.

### 4.3. Investigation of the Growth Pattern of Representative Leaf Blades of S. kogasensis ‘Aureostriatus’

First, 30 leaves of *S. kogasensis* ‘Aureostriatus’ were marked and blade elongation was measured once a day from the appearance of the leaf tip until the leaf was fully expanded. At the same time, another 30 representative leaves as the same developmental stage were collected, brought back to the laboratory, and immersed in water to spread the leaf blades and measure their width and thickness. In addition, we collected 20 young leaves each day at different developmental stages (leaf blades without leaf sheaths) and measured the length of the leaves.

### 4.4. Cytological Observations of Leaf Epidermal and Mesophyll Cells during the Period of Rapid Elongation

Leaves measuring 0.1, 0.5, 1, 3, 11, 19, and 21 cm were chosen as the materials for the microscopic analysis, including incompletely expanded and mature leaves, with five leaves in each period. Young leaves were divided into units of 0.5 cm, while those 11 cm and longer were divided into 1 cm units and numbered from base to tip. The samples were fixed in 50% formalin–acetic acid (FAA) alcohol buffer and transferred to 70% FAA buffer for long-term preservation after two days. The mature tissues were preserved by fixing them in 70% FAA buffer. Leaf samples measuring 10 × 5 mm were collected from the center of each unit. These samples were then rehydrated by immersing them in a gradient of ethanol concentrations (50%, 30%, 15%, and pure water). Subsequently, the samples were desilicated using a 25% hydrofluoric acid solution (young tissues (0.1, 0.5, 1 and 3 cm) were not siliconized) [70]. The samples were successively immersed in ethanol solutions of different concentrations (15%, 30%, and 50%) and xylene solutions. The paraffin was then crushed and placed in the xylene solution before being left in an oven at 38 °C overnight. Longitudinal and transverse sections of bamboo leaves, with a thickness of 7 μm, were obtained by slicing paraffin-embedded tissues using a Leica RM 2235 rotary microtome. The sections were then deparaffinized and stained sequentially with safranin and Fast Green and observed using a Leica DM2500 light microscope (Wetzlar, Germany).

Four tissue sections were selected from each sample, and five microscopic fields of view were randomly chosen per section to determine the cell size. Each cell type’s length, width, and thickness (epidermis and mesophyll) within each field of view were manually measured using the Leica DM2500 light microscope software (Leica Application Suite X, Leica, Wetzlar, Germany). Measurements were taken for 50–100 cells of each type.

### 4.5. Observation of the Microstructure of the Leaf Epidermis during Rapid Elongation

The leaf blades in the rapid elongation stage were cut into 0.5 × 0.5 cm squares, with 1 cm as the unit, from the base to the tip of each blade. Subsequently, the leaf squares were soaked in a 30% hydrogen peroxide–acetic acid solution for 6 h at 65 °C. The epidermis was separated from the macerated mesophyll (including veins) and rinsed with distilled water. Next, the epidermis was stained with a 2% safranin solution for 5 min. The microstructure of the stained leaf epidermis was then observed using a Leica DM2500 microscope (Leica, Wetzlar, Germany).

### 4.6. Data Analysis and Graphics

Data statistics processes were performed using Excel and SPSS 17. Differences between datasets from different growth stages were compared using a one-way ANOVA using IBM SPSS software (R23.0). Images from the processed data were generated using GraphPad Prism 8.

## 5. Conclusions

**Figure 8:**
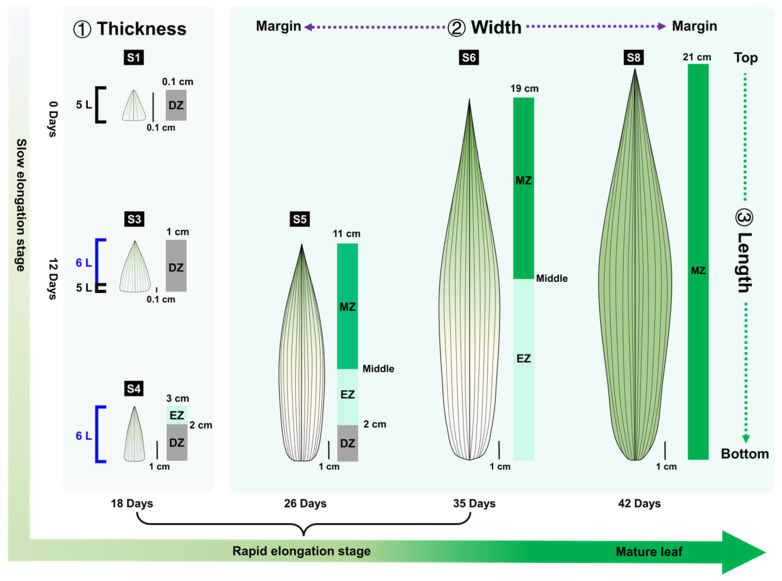
This anatomical model was developed for the growth and development of the leaves of *S. kogasensis* ‘Aureostriatus’. Note: DZ, division zone; EZ, elongation zone; L, cell layer count; MZ, mature zone.

In conclusion, we propose a three-dimensional growth model for typical leaves of *S. kogasensis* ‘Aureostriatus’ (Figure 8). Our findings reveal variations in the distribution of leaf growth zones during rapid elongation at different developmental stages. The meristematic tissue cells positioned at the leaf base remained uniform in size and spatial distribution during steady-state elongation, while the anisotropy of the cell division was most notable in this area. Additionally, the findings indicate a noteworthy correlation between the longitudinal growth of leaves and the expansion of epidermal and mesophyll cells. Moreover, the increases in leaf width and thickness are facilitated by divisions in meristematic tissues and the expansion of post-divisional cells. These results provide a framework for understanding the regulatory mechanism responsible for the rapid elongation of leaves in *S. kogasensis* ‘Aureostriatus’.

## Data Availability

Data is contained within the article.
